# Redox status of apolipoprotein E in cerebrospinal fluid: a mechanistically informative biomarker for central nervous system disorders

**DOI:** 10.1042/BSR20250388

**Published:** 2026-05-14

**Authors:** Yui Uematsu, Wakana Iinuma, Riho Shimizu, Hiroto Matsuura, Tsuneaki Yoshinaga, Masahide Yazaki, Kazuyoshi Yamauchi

**Affiliations:** 1 Department of Clinical Laboratory Investigation, Graduate School of Medicine, Shinshu University, Matsumoto, Japan; 2 Department of Laboratory Medicine, Shinshu University Hospital, Matsumoto, Japan; 3 Department of Neurology and Rheumatology, Shinshu University, Matsumoto, Japan; 4 Division of NeuroHealth Innovation, Institute for Biomedical Sciences, Shinshu University, Matsumoto, Japan; 5 Department of Biomedical Laboratory Sciences, School of Health Sciences, Shinshu University, Matsumoto, Japan

**Keywords:** Alzheimer’s disease, cholesterol transport, Cysteine-thiol, disulfide bond, oxidative stress

## Abstract

Apolipoprotein (apo) E is the major cholesterol carrier in the central nervous system (CNS); however, the clinical relevance of its cysteine-thiol redox status in cerebrospinal fluid (CSF) remains unclear. We investigated whether CSF apoE redox indices (redox-IDX-apoE) reflect cholesterol transport efficiency and disease-specific pathologies. We quantified reduced (red), reversibly oxidized (roxi), and irreversibly oxidized (oxi) apoE in CSF and serum using a maleimide-based band-shift assay. We analyzed relationships between redox-IDX-apoE, CSF cholesterol (TC) level, and the TC/apoE ratio (inverse transport efficiency) in patients with apoE3/E3 and identified transport determinants using isometric log-ratio (ILR) regression. Significant but only moderate correlations between CSF and serum indices suggested distinct redox behavior in the two compartments. ApoE3/E4 carriers exhibited higher oxi-apoE, reflecting reduced buffering capacity. In apoE3/E3 CSF, aging increased roxi/total and decreased red/roxi, suggesting a shift toward oxidized forms. CSF TC levels positively correlated with roxi-related indices. Conversely, the TC/apoE ratio negatively correlated with red/roxi, indicating that red-apoE supports higher efficiency. ILR analysis confirmed that maintaining the reduced monomeric state, rather than the reversibly oxidized form, was independently associated with improved transport efficiency. Diagnostic groups exhibited distinct signatures: neurodegenerative disorders showed elevated irreversible oxidation, whereas neuroimmunological and infectious conditions exhibited profiles suggestive of reversible and acute oxidation, respectively. The CSF apoE redox status links local redox balance to cholesterol handling and reflects CNS pathophysiology. Maintaining reduced cysteine-thiol appears important for functional capacity, whereas a shift toward oxidation reflects a trade-off between buffering ability and transport efficiency. These indices may serve as potential biomarkers.

## Introduction

Apolipoprotein (apo) E, a 35-kDa glycoprotein comprising 299 amino acids, is primarily produced in the liver and plays a pivotal role in cholesterol homeostasis and immune regulation in systemic circulation [1,2]. ApoE is also expressed in the brain, which is the second largest site of apoE production [3], and is a major component of lipoproteins in the cerebrospinal fluid (CSF) [4]. Similar to the function of plasma apoE, cerebral apoE is involved in cholesterol transport and metabolism and immune regulation, thereby contributing to the growth and maintenance of the central nervous system (CNS) [5,6].

Human apoE exists in three major isoforms—apoE2, apoE3, and apoE4—which are encoded by three common alleles at a single genetic locus [7]. These isoforms differ by a cysteine (Cys)–arginine (Arg) interchange at residues 112 and/or 158 (apoE2, Cys112/Cys158; apoE3, Cys112/Arg158; apoE4, Arg112/Arg158) [8]. Despite involving only single amino acid substitutions, these variations markedly affect the structure and function of each isoform, leading to various pathological outcomes [2,9]. Numerous studies have demonstrated that apoE polymorphisms are closely associated with the pathogenesis of various disorders in both the periphery and CNS [5,9]. Notably, apoE4 is the strongest genetic risk factor for sporadic late-onset Alzheimer’s disease (AD) [10] and is associated with an increased incidence and earlier onset [11]. However, the precise role of apoE in the pathogenesis of AD remains unclear.

Given that a substantial proportion of patients with AD are non-carriers of the apoE4 allele [12], it is essential not only to elucidate the mechanisms underlying the detrimental effects caused by apoE4 but also to gain further insight into the physiological and pathological roles of apoE2 and apoE3. Therefore, in the present study, we focused on the Cys residues, which not only differentiate the isoforms but also determine their specific redox properties [13–17].

The thiol group of Cys is a highly reactive and functionally important moiety that imparts specific properties to proteins depending on their redox state [18,19]. This post-translational modification regulates a variety of physiological processes, including signaling pathways, transcriptional modulation, protein maturation, and defense against oxidative stress [20–22]. Notably, irreversible oxidation to sulfonic acid leads to the loss of function and degradation of the modified protein [23,24], whereas reversible oxidations, such as disulfide bond formation, act as a protective redox buffer rather than resulting in permanent damage [21,25]. In the context of apoE, disulfide-linked complexes, including homodimers, apoE–AII complexes [26], and apoAII-E2–AII complexes [27], represent reversibly oxidized forms of apoE2 and apoE3. We previously reported that these disulfide-linked complexes contribute to the maintenance of the redox balance of apoE by preventing irreversible oxidation [14] and enhancing its interaction with liposomes [15]. Furthermore, we proposed that redox indices of Cys-thiol of serum apoE, which are determined by calculating the values of the reduced form (red-), reversibly oxidized form (roxi-), irreversibly oxidized form (oxi-), and total apoE, may serve as diagnostic markers for oxidative stress–driven diseases, such as atherosclerosis [16].

Oxidative stress induces inflammation and disrupts lipid homeostasis, ultimately contributing to the formation of atherosclerotic lesions [28]. Similar mechanisms are implicated in the development of sporadic late-onset AD [29]. The CNS is particularly susceptible to oxidative damage because of its high oxygen consumption and abundant lipid content [30]. There is increasing evidence indicating that immune dysregulation and altered cholesterol metabolism in the CNS play key roles in AD pathogenesis [31,32]. Given that apoE serves as a critical regulator of both lipid transport and immune modulation, we hypothesized that the redox status of CNS apoE is a key determinant of AD pathology.

To test this hypothesis, we aimed to evaluate the clinical relevance of redox status of apoE in the CSF and explore its potential as a novel biomarker for CNS disorders. Specifically, we assessed the redox status of CSF apoE using a previously developed band-shift assay with photocleavable maleimide-conjugated polyethylene glycol (PEG-PC-Mal) [13].

## Materials and methods

### Materials

Horseradish peroxidase (HRP)-conjugated goat anti-apoE polyclonal antibody was purchased from Fortis Life Sciences (Boston, MA, U.S.A.). PEG-PC-Mal was obtained from Dojindo Molecular Technologies Inc. (Kumamoto, Japan). All other chemicals and reagents were of analytical grade.

### Subjects

In total, 105 participants were enrolled in the present study. CSF samples were obtained via lumbar puncture. To ensure the absence of blood contamination, each sample was examined microscopically, and only those with no detectable red blood cells were included in the study. Paired serum samples were collected within five days of CSF sampling. To prevent artifactual oxidation caused by repeated freeze–thaw cycles, all samples were aliquoted in small volumes and stored at −80°C until analysis. The clinical characteristics of the study participants are summarized in Table 1. Based on clinical diagnoses, the participants were classified into five categories: neurodegenerative disorders (G1), neuroimmunological/inflammatory disorders (G2), infectious diseases (G3), other conditions (G4), and tumor-related disorders (G5). Specific diagnoses of patients included in each group are listed in Supplementary Table S1. The present study was conducted in accordance with the principles of the Declaration of Helsinki and was approved by the Ethical Review Board of Shinshu University School of Medicine (approval numbers: 6015 and 6487). Written informed consent was obtained from all participants or their legal representatives.

**Table 1:** Characteristics of the present study subjects

Variables	Total	G1	G2	G3	G4	G5
n (female / male)	105 (56/49)	21 (11/10)	25 (18/7)	9 (3/6)	22 (11/11)	28 (14/14)
Age, years						
mean ± SD	51.7 ± 22.9	67.9 ± 12.0	50.2 ± 16.9	51.1 ± 14.8	61.0 ± 8.5	62.2 ± 9.1
apoE phenotype, n (%)						
E2/E3	8 (7.6)	−	1 (4.0)	−	3 (13.6)	4 (14.3)
E2/E4	2 (1.9)	−	−	−	1 (4.5)	1 (3.6)
E3/E3	80 (76.2)	18 (85.7)	20 (80.0)	7 (77.8)	15 (68.2)	20 (71.4)
E3/E4	14 (13.3)	30 (14.3)	3 (12.0)	2 (22.2)	3 (13.6)	3 (10.7)
E4/E4	1 (1.0)	−	1 (4.0)	−	−	−

G1, neurodegenerative disorders; G2, neuroimmunological/inflammatory disorders; G3, infectious diseases; G4, other conditions; G5, tumor-related disorders.

### Determination of apoE redox status

The redox status of apoE was analyzed using a band-shift assay with PEG-PC-Mal, as described in our previous study [13]. Briefly, PEG-PC-Mal was added to a serum or CSF sample at a final concentration of 1.0 mmol/l, followed by incubation for 30 min at 37°C. Serum samples were diluted 20-fold with saline prior to the reaction. Subsequently, 10 μl of each sample was mixed with non-reducing Laemmli buffer [33] and subjected to 8–16% gradient sodium dodecyl sulfate–polyacrylamide gel electrophoresis. After electrophoresis, the gel was irradiated with ultraviolet light for 15 min to cleave the PEG moiety and the separated proteins were transferred onto a polyvinylidene fluoride membrane. The membrane was then probed with an HRP-conjugated anti-apoE polyclonal antibody. The specific bands were visualized using an enhanced chemiluminescence detection reagent and quantified using ImageJ 1.45 software (National Institutes of Health, Bethesda, MD, U.S.A.).

### Determination of apoE and total cholesterol

The levels of serum and CSF apoE were determined using a turbidimetric assay with the ApoE Auto N DAIICHI (Sekisui Medical Co., Ltd., Tokyo, Japan). CSF total cholesterol levels were determined using the cholesterol oxidase method (Canon Medical Diagnostics Co., Tokyo, Japan). Both analyses were performed using a JCA-ZS050 automated analyzer (JEOL, Ltd., Tokyo, Japan). For the determination of CSF apoE and TC, the assay conditions were modified by increasing the sample-to-reagent volume ratio to 20-fold higher than that used for serum samples. The analytical performance of these modified assays for CSF samples (e.g., linearity and precision) was validated prior to the study (Supplementary Figure S1 and Supplementary Table S2). These validation results confirmed their suitability for the quantitative evaluation of apoE redox status in the subsequent analyses.

### ApoE phenotyping

The apoE phenotype of all participants was determined using serum samples by isoelectric focusing and immunoblot analysis, as previously described [34].

### Statistical methods

Continuous variables are presented as means ± standard deviation (SD) for normally distributed data and as medians (interquartile range: 25th–75th percentile) for non-normally distributed data. Differences between two groups were assessed using the Mann–Whitney *U* test. Results from three or more groups were analyzed using the Kruskal–Wallis test with the Steel–Dwass post hoc test. Sex distribution was compared using the chi-square test. Correlations were evaluated using the Spearman’s rank correlation coefficient. Determinants of transport efficiency were analyzed using isometric log-ratio (ILR) regression analysis. Statistical significance was set at *P* <0.05. All statistical analyses were performed using Bell Curve for Excel (Social Survey Research Information Co., Ltd., Tokyo, Japan).

## Results

### Typical band-shift assay patterns associated with CSF apoE redox status

**Figure 1:**
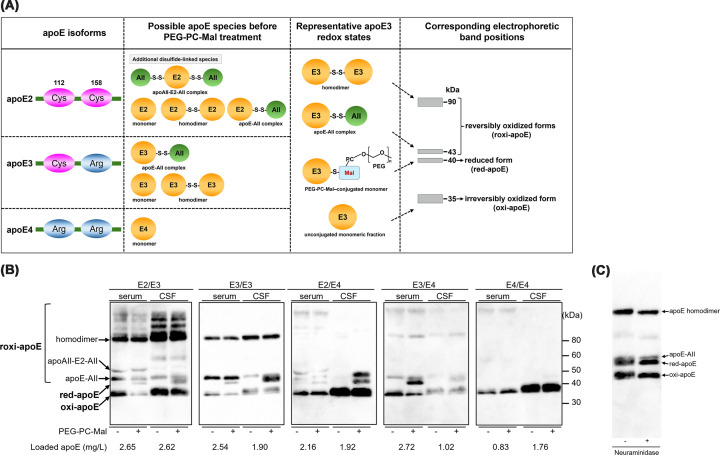
Characterization of apoE redox status in CSF and serum using a band-shift assay Schematic overview of the cysteine content of each apoE isoform and the corresponding interpretation of apoE redox states in the PEG-PC-Mal band-shift assay. (**A**) The two panels on the right illustrate representative apoE3 redox states after PEG-PC-Mal treatment and their corresponding electrophoretic band positions. Representative western blotting images of apoE in the serum and CSF of participants with different apoE phenotypes (E2/E3, E3/E3, E2/E4, E3/E4, and apoE4/E4). (**B**) The samples were then incubated with or without PEG-PC-Mal. The 40-kDa band shifted by PEG-PC-Mal was defined as the reduced form (red-apoE). The remaining unmodified monomeric band was defined as the irreversibly oxidized form (oxi-apoE). Higher-molecular-weight bands, including homodimers and heterodimers, were defined as reversibly oxidized forms (roxi-apoE). The total apoE concentrations of the samples loaded onto the gel (using undiluted CSF and 20-fold diluted serum) are indicated below each lane (mg/l). A consistent volume of 10 μl was applied to each lane. The bracket indicates the range of apoE-containing bands quantified as roxi-apoE, comprising all species migrating above the red-apoE position. Effect of neuraminidase treatment on the electrophoretic mobility of CSF apoE (**C**).

A schematic overview of the cysteine content of each apoE isoform and the corresponding interpretation of apoE redox states in the PEG-PC-Mal band-shift assay is shown in Figure 1A. The representative band-shift assay patterns of CSF apoE were fundamentally similar to those observed in serum apoE (Figure 1B). In participants with apoE3/E3 and apoE3/E4, incubation with PEG-PC-Mal induced a new 40-kDa band in both serum and CSF, in addition to the bands corresponding to the 35-kDa monomer, homodimer, and the heterodimeric apoE3–AII complex. In those with apoE2-containing phenotypes (apoE2/E3 and apoE2/E4), additional bands, including the heterodimeric apoAII-E2–AII complex and several bands with a molecular weight higher than that of the homodimer, were observed in both compartments (Figure 1B). Based on our previous studies [13,16], the bands were defined as follows: the 40-kDa band corresponding to a single PEG-PC-Mal adduct was defined as the reduced form (red-apoE); the PEG-PC-Mal–unconjugated monomer that remained despite incubation with PEG-PC-Mal was termed the irreversibly oxidized form (oxi-apoE). This fraction is operationally defined as oxi-apoE based on its non-reactivity with the maleimide reagent; however, it should be noted that this fraction may potentially include a minor portion of reduced thiols where the sulfhydryl group is sterically inaccessible. Other bands, including homodimers, heterodimers, and their PEG-PC-Mal conjugates, were collectively termed reversibly oxidized forms (roxi-apoE). Notably, CSF red- and oxi-apoE exhibited broad, slightly higher-molecular-weight migration patterns than those of serum. Neuraminidase digestion abrogated this size shift and sharpened the bands (Figure 1C).

### Comparison of redox-IDX-apoE between CSF and serum

To comprehensively evaluate the redox status of apoE, we defined several indices, termed redox-IDX-apoE, which reflect the equilibrium among the reduced, reversibly oxidized, and irreversibly oxidized forms, as well as the distribution between functional and dysfunctional apoE (Table 2). To determine these indices, the concentrations of red- and oxi-apoE were calculated by multiplying their respective fractions by the total apoE concentration. The roxi-apoE concentration was calculated by subtracting the sum of the red- and oxi-apoE concentrations from the total apoE concentration. The redox-IDX-apoE was determined based on these values. A comparative analysis between CSF and serum revealed significant but moderate correlations for most redox-IDX-apoEs (Supplementary Table S3).

**Table 2:** Redox-IDX-apoE

Index	Description
red/total	Ratio of reduced apoE (red-apoE) to total apoE
roxi/total	Ratio of reversibly oxidized apoE (roxi-apoE; homodimer + heterodimers) to total apoE
oxi/total	Ratio of irreversiblly oxidized apoE (oxi-apoE) to total apoE
oxi/red	Ratio of oxi-apoE to red-apoE
oxi/roxi	Ratio of oxi-apoE to roxi-apoE
oxi/(red + roxi)	Ratio of dysfunctional apoE (oxi-apoE) to functional apoE (red-apoE + roxi-apoE)
roxi/(red + oxi)	Ratio of polymeric apoE (roxi-apoE) to monomeric apoE (red-apoE + oxi-apoE)
red/roxi	Ratio of red-apoE to roxi-apoE
red/(oxi + roxi)	Ratio of red-apoE to total oxidized apoE (oxi-apoE + roxi-apoE)

### Effect of apoE phenotype on CSF redox-IDX-apoE

**Figure 2:**
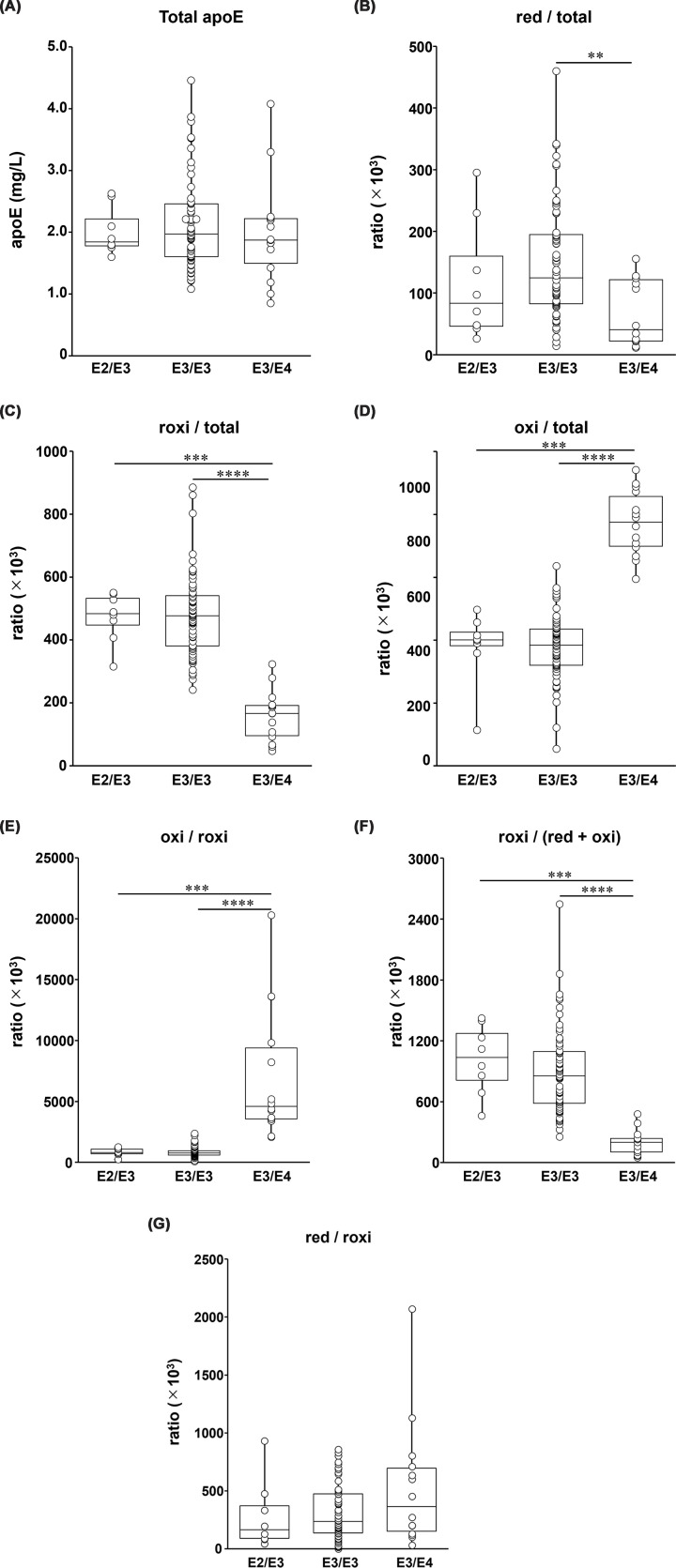
Impact of apoE phenotype on redox indices in CSF Comparison of CSF apoE redox indices among participants with different phenotypes: E2/E3 (*n* = 8), E3/E3 (*n* = 80), and E3/E4 (*n* = 14). The concentrations of total apoE (**A**) and the ratios of red/total (**B**), roxi/total (**C**), and oxi/total (**D**), oxi/roxi (**E**), roxi/(red + oxi) (**F**), and red/roxi (**G**) are shown. Data are presented as box-and-whisker plots (medians and interquartile ranges) with individual data points overlaid (jittered). Statistical differences were analyzed using the Kruskal–Wallis test, followed by the Steel–Dwass post hoc test. *P <0.05, **P <0.01, ***P <0.001, ****P <0.0001.

Next, we examined the effect of the apoE phenotype, specifically the number of Cys residues per two apoE molecules, on CSF redox-IDX-apoE. However, the apoE4/E4 phenotype was not included in this analysis because apoE4 lacks Cys residues, and participants with apoE2/E4 (*n* = 2) were excluded because of the very small sample size. Therefore, comparisons were performed among the apoE2/E3, apoE3/E3, and apoE3/E4 groups. To facilitate interpretation, we first focused on the primary fractions (total apoE, red/total, roxi/total, and oxi/total) and then examined selected derived ratios as complementary indicators of the redox balance. Although there was no significant difference in total apoE concentration among the three different apoE phenotype groups (Figure 2A), the red/total and roxi/total ratios tended to decrease with a decrease in the total number of Cys residues (Figure 2B,C), whereas the oxi/total ratio showed the opposite trend (Figure 2D). Specifically, the red/total ratio in participants with apoE3/E4 was markedly lower than that in those with apoE3/E3 (approximately 0.3-fold, *P* <0.01). The roxi/total ratio in participants with apoE3/E4 was also significantly lower than that in those with apoE3/E3 or apoE2/E3 (approximately 0.2-fold; *P* <0.0001 versus E3/E3; *P* <0.001 versus E2/E3). Conversely, the oxi/total ratio was significantly higher in participants with apoE3/E4 than in those with apoE3/E3 or apoE2/E3 (approximately 2.3-fold; *P* <0.0001 versus E3/E3; *P* <0.001 versus E2/E3). Consistent with these primary changes, the derived ratios also indicated a shift toward an oxi-dominant redox profile in participants with apoE3/E4. All ratios with oxi in the numerator, namely oxi/red and oxi/(red + roxi) (Supplementary Figure S2a, b), as well as oxi/roxi (Figure 2E), were significantly higher in participants with apoE3/E4 than in the other two groups. Specifically, the oxi/red ratio was approximately 4.6-fold higher than in participants with E3/E3 (*P* <0.0001) and E2/E3 (*P* <0.05). The oxi/roxi and oxi/(red + roxi) ratios showed even more pronounced elevations, ranging from 7.1- to 8.5-fold, compared with the two other groups (*P* <0.0001 versus E3/E3; *P* <0.001 versus E2/E3). In contrast, the roxi/(red + oxi) ratio was significantly lower in participants with apoE3/E4 than in other groups (approximately 0.2-fold; *P* <0.0001 versus E3/E3, *P* <0.001 versus E2/E3) **(**Figure 2F). The red/(oxi + roxi) ratio was also significantly lower in participants with apoE3/E4 than in those with apoE3/E3 (approximately 0.4-fold, *P* <0.01). It was also lower than that in those with apoE2/E3 (0.5-fold); however, the difference was not statistically significant (Supplementary Figure S2c). No significant differences in red/roxi were observed among the three apoE phenotypes (Figure 2G). Taken together, these findings indicate that the redox status of apoE is strongly influenced by the number of available Cys residues and varies substantially across apoE phenotypes. Therefore, to minimize the confounding effect of the Cys residue number, subsequent analyses were restricted to participants with the apoE3/E3 phenotype.

### Effect of age on CSF redox-IDX-apoE

**Figure 3:**
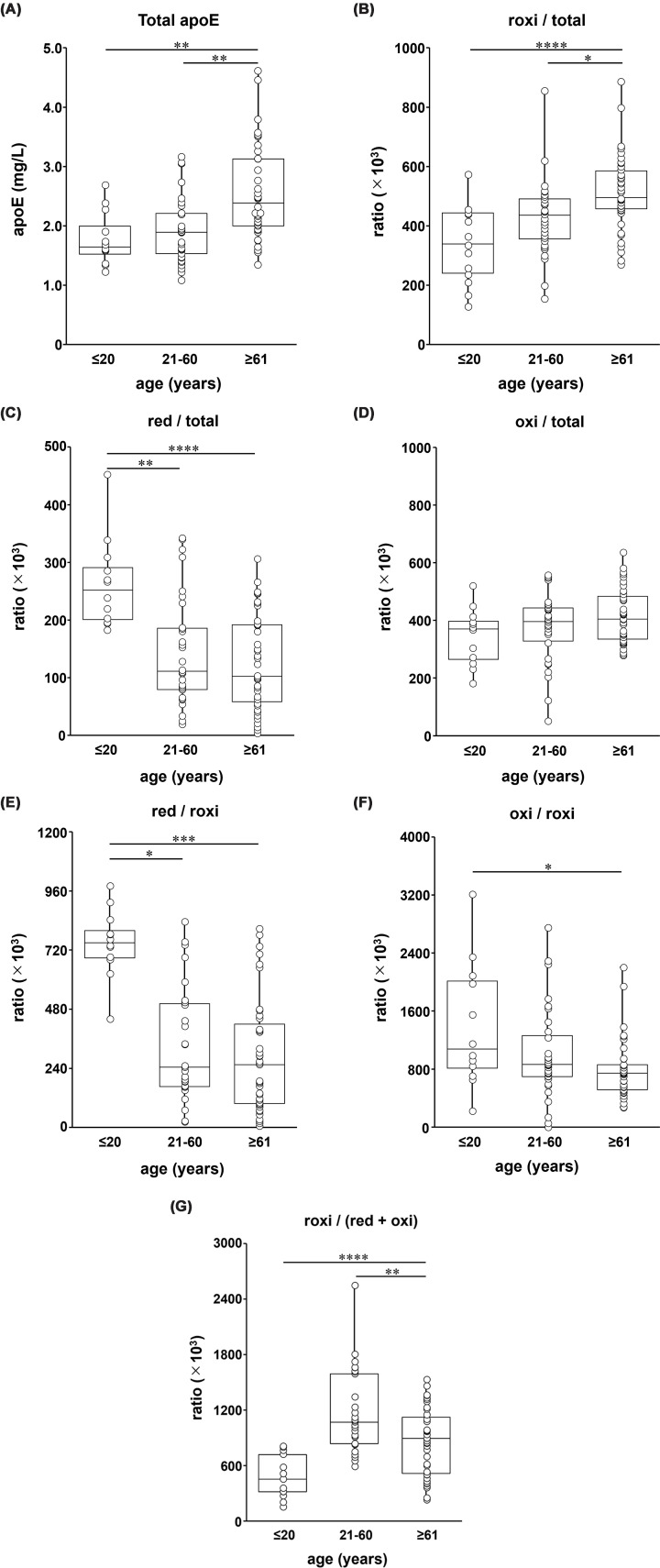
Age-related changes in CSF apoE redox status in participants with apoE3/E3 Participants with the apoE3/E3 phenotype were classified into three age groups: young-age (≤20 years, *n* = 12), middle-age (21–60 years, *n* = 32), and old-age (≥61 years, *n* = 36). Concentrations of total apoE (**A**) and the ratios of roxi/total (**B**), red/total (**C**), red/roxi (**D**), oxi/roxi (**E**), roxi/(red + oxi) (**F**), and oxi/total (**G**) are shown. Data are presented as box-and-whisker plots (medians and interquartile ranges) with individual data points overlaid (jittered). Statistical differences were analyzed using the Kruskal–Wallis test followed by the Steel–Dwass post hoc test. *P <0.05, **P <0.01, ***P <0.001, ****P <0.0001.

To assess age-related changes in the CSF apoE redox status, we analyzed participants with the apoE3/E3 phenotype, stratified into three groups based on age: young-age (≤20 years), middle-age (21–60 years), and old-age (≥61 years). The individual ages of the participants in each age group are shown in Supplementary Figure S3, and the biochemical characteristics of these age-defined cohorts, including TC, total apoE, and the red, roxi, and oxi forms of apoE, are summarized in Supplementary Table S4. To facilitate interpretation, we first focused on the primary fractions (total apoE, red/total, roxi/total, and oxi/total) and then examined selected derived ratios as complementary indicators of age-related shifts in the redox balance. Overall, aging was characterized by an increase in total apoE and the reversibly oxidized fraction, together with a decline in the reduced fraction, whereas oxi/total showed no significant overall change. The total apoE concentration and roxi/total ratio increased significantly with age (Figure 3A,B). Specifically, the total apoE concentration in the old-age group was approximately 1.4-fold higher than that in both the middle- and young-age groups (*P* <0.01). The roxi/total ratio in the old-age group was also significantly higher than that in the young-age (approximately 1.5-fold, *P* <0.0001) and middle-age (approximately 1.2-fold, *P* <0.05) groups. By contrast, red/total was significantly lower in the middle-age and old-age groups than in the young-age group (approximately 0.5-fold; *P* <0.01 for middle-age versus young-age, and *P* <0.0001 for old-age versus young-age) (Figure 3C), whereas oxi/total did not differ significantly among the three age groups (Figure 3D). Consistent with these primary changes, several derived ratios also supported an age-related shift in the redox balance. Although the difference was not statistically significant, the oxi/red ratio in the old-age group was also approximately 2.8-fold higher than that in the young-age group and 1.5-fold higher than that in the middle-age group (Supplementary Figure S4a). Likewise, red/(oxi + roxi) was significantly lower in the middle-age and old-age groups (approximately 0.7-fold for middle-age versus young-age, *P* <0.05; and 0.5-fold for old-age versus young-age, *P* <0.001) (Supplementary Figure S4b). Furthermore, red/roxi showed a progressive decline and was significantly lower in the middle-age (approximately 0.5-fold, *P* <0.05) and old-age groups (approximately 0.3-fold, *P* <0.001) than in the young-age group (Figure 3E). The oxi/roxi ratio was also significantly lower in the old-age group than in the young-age group (approximately 0.6-fold, *P* <0.05) (Figure 3F). Notably, the roxi/(red + oxi) ratio exhibited a nonlinear pattern with the highest value being noted in the middle-age group (Figure 3G). Specifically, the ratio in the middle-age group was approximately 2.4-fold that in the young-age group (*P* <0.0001) and 1.4-fold that in the old-age group (*P* <0.05). No significant differences were observed in oxi/(red + roxi) among the three age groups (Supplementary Figure S4c). Visual inspection of the band-shift patterns suggested that the age-related increase in roxi-apoE involved both homodimeric and heterodimeric species, although individual heterodimeric bands were not consistently resolved enough for separate quantification. Taken together, these findings reveal a progressive age-dependent shift in the CSF apoE redox equilibrium toward a more oxidized state, characterized by a significant decline in the reduced monomeric form alongside an increase in reversible oxidation and total apoE concentration.

### Effect of CSF apoE redox status on CSF TC levels and TC/apoE ratio

For reference, the absolute concentrations of CSF TC and the TC/apoE ratios stratified by apoE phenotype are summarized in Table 3. No significant differences in CSF TC or TC/apoE were observed among the analyzed apoE phenotype groups. However, to minimize confounding by apoE phenotype, as in the age-stratified analysis, the following analyses were restricted to participants with apoE3/E3. Significant associations between CSF TC levels and apoE redox status were observed in participants with apoE3/E3. Specifically, CSF TC levels showed positive correlations with total apoE (ρ = 0.376, *P* <0.0001), roxi/total (ρ = 0.378, *P* <0.001), and roxi/(red + oxi) (ρ = 0.389, *P* <0.001). In contrast, negative correlations were observed with red/total (ρ = −0.229, *P* <0.05), oxi/total (ρ = −0.233, *P* <0.05), oxi/roxi (ρ = −0.326, *P* <0.01), red/roxi (ρ = −0.321, *P* < 0.01), oxi/(red + roxi) (ρ = −0.221, *P* <0.05), and red/(oxi + roxi) (ρ = −0.231, *P* <0.05) (Table 4). However, CSF TC levels reflect multiple processes, including production, exchange, and clearance; therefore, they may not directly represent apoE-mediated cholesterol transport efficiency.

**Table 3:** CSF total cholesterol (TC) concentrations and TC/apoE ratios across apoE phenotypes

apoE phenotype	n	apoE (mg/l)	CSF TC (mg/l)	TC/apoE
E2/E3	8	1.89 (1.77–2.34)	3.22 (3.16–4.57)	1.80 (1.46–2.09)
E3/E3	80	1.95 (1.60–2.46)	3.33 (2.79–4.06)	1.51 (1.21–2.08)
E3/E4	14	1.88 (1.42–2.23)	3.58 (2.90–4.37)	1.68 (1.24-2.44)

Data are presented as median (interquartile range). The apoE2/E4 and apoE4/E4 phenotypes were excluded because apoE4/E4 lacks Cys residues and the number of participants with apoE2/E4 was very small (*n* = 2).

**Table 4:** Spearman’s rank correlation coefficients between apoE redox indices and cholesterol parameters in apoE3/E3 subjects

Redox indices	CSF TC levels		TC/apoE ratio	
	ρ	*P*	ρ	*P*
red/total	−0.229	**0.0362**	−0.469	**8.90 × 10^−6^**
roxi/total	0.378	**3.9 × 10^−4^**	0.251	**0.0213**
oxi/total	−0.233	**0.0333**	0.117	0.2880
oxi/red	0.104	0.3440	0.410	**0.0001**
oxi/roxi	−0.326	**0.0025**	−0.057	0.6060
red/roxi	−0.321	**0.0029**	−0.451	**2.10 × 10^−6^**
oxi/(red + roxi)	−0.221	**0.0433**	0.131	0.2340
red/(oxi + roxi)	−0.231	**0.0348**	−0.467	**9.81 × 10^−6^**
roxi/(red + oxi)	0.389	**2.6 × 10^−4^**	0.262	**0.0164**
Total apoE	0.376	**4.2 × 10^−4^**	−0.449	**1.89 × 10^−5^**

Bold values indicate statistical significance (*P* <0.05).

To further explore the functional implications of apoE redox status, we evaluated its influence on lipid transport efficiency. In this analysis, the TC/apoE ratio, which represents the cholesterol load per apoE molecule, was used as an inverse proxy for cholesterol transport efficiency; a higher TC/apoE ratio suggests impaired cholesterol release by apoE. The TC/apoE ratio was positively correlated with roxi/total (ρ = 0.251, *P* <0.05), oxi/red (ρ = 0.410, *P* <0.001), and roxi/(red + oxi) (ρ = 0.262, *P* <0.05); however, it was negatively correlated with total apoE (ρ = −0.449, *P* <0.0001), red/total (ρ = −0.469, *P* <0.0001), red/roxi (ρ = −0.451, *P* <0.001), and red/(oxi + roxi) (ρ = −0.467, *P* <0.0001) (Table 4). These correlational data indicate that the redox state of CSF apoE is significantly associated with cholesterol homeostasis, with a shift from the reduced monomeric form toward oxidized species—whether reversible or irreversible—correlating with reduced lipid transport efficiency, as reflected by an elevated TC/apoE ratio.

### Identification of apoE redox-based determinants of cholesterol transport

To determine the independent drivers of cholesterol transport capacity while accounting for the compositional interdependence of apoE redox species (constrained by a constant sum), we performed multivariable regression analysis using ILR coordinates in participants with apoE3/E3. The log-transformed TC/apoE ratio, serving as an inverse proxy for transport efficiency, was modeled as the dependent variable, adjusting for age and sex. Overall, two ILR coordinates were constructed to represent distinct oxidation axes: *ILR_1_* [log(oxi/(red + roxi))], reflecting the balance between irreversible oxidation and the reversible/reduced pool; and *ILR_2_* [log(red/roxi)], representing the shift within the reversible pool toward the reduced state.

**Figure 4:**
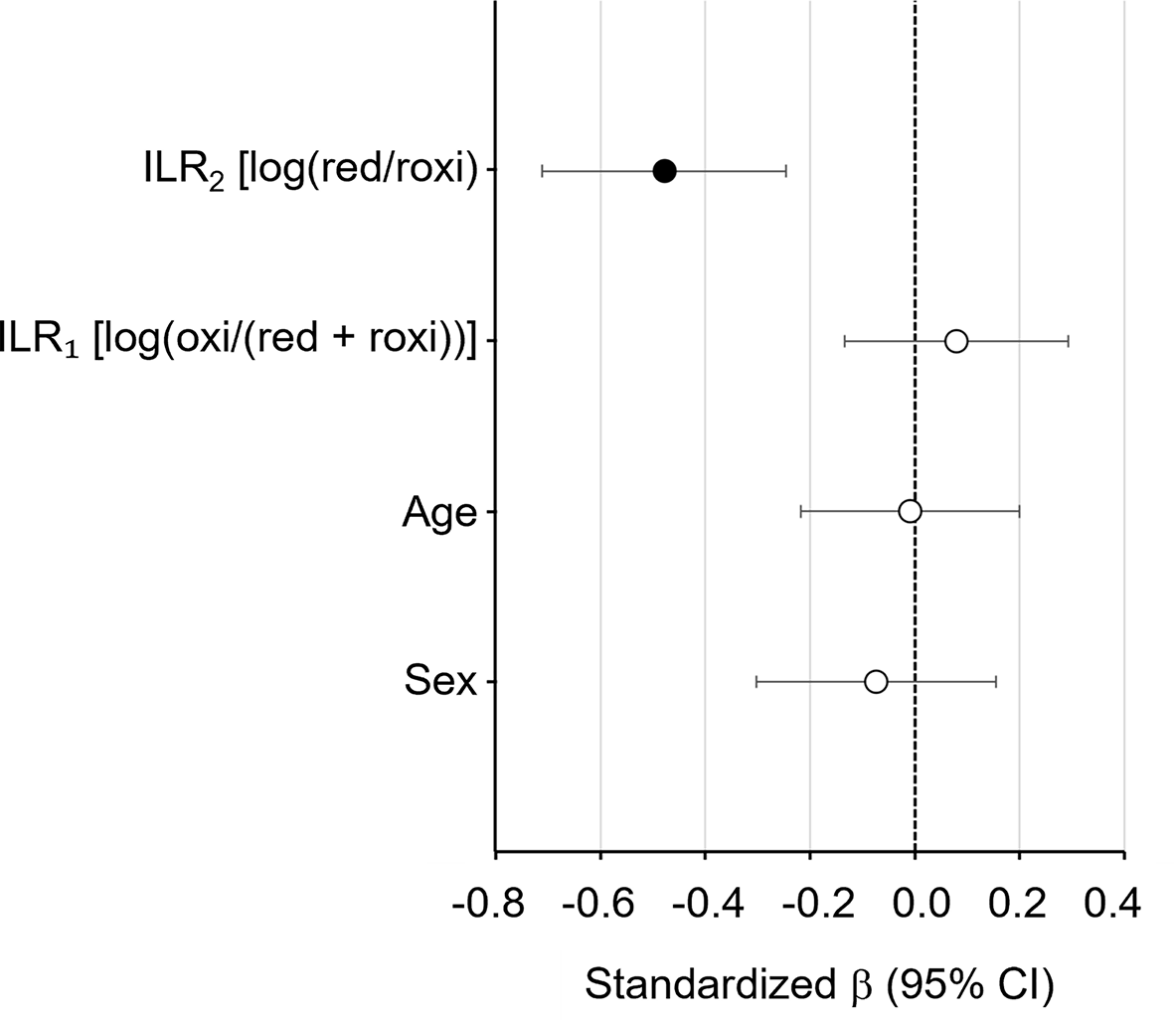
Forest plot of ILR model determinants for cholesterol transport efficiency Forest plot showing standardized regression coefficients (β) and 95% confidence intervals from the multivariable ILR regression model for log(TC/apoE). Independent variables included sex (male = 0, female = 1), age, *ILR_1_* [log(oxi/(red + roxi))], and *ILR_2_* [log(red/roxi)]. Variance inflation factors (VIF) confirmed the absence of multicollinearity (VIF < 5).

*ILR_2_* exhibited a significant negative association with the log(TC/apoE) ratio (Standardized *β* = −0.479, *P* <0.0001). This indicated that a relative increase in the reduced monomer (red-apoE) over the reversibly oxidized dimer (roxi-apoE) was independently correlated with lower TC/apoE ratios (i.e., higher transport efficiency). In contrast, *ILR_1_* showed no significant association with the log(TC/apoE) ratio (standardized *β* = 0.079, *P* = 0.462). These findings suggest that the equilibrium of the reversible oxidation axis (*ILR_2_*), rather than the irreversible oxidation burden (*ILR_1_*), is the primary determinant of apoE-mediated lipid transport efficiency in the CNS (Figure 4 and Table 5).

**Table 5:** Multivariable ILR regression analysis determining predictors of cholesterol transport efficiency (log[TC/apoE])

Variables	B	SE	β	95% CI	*t*	*P*	VIF
Sex	−0.014	0.157	−0.009	−0.326, 0.299	−0.087	0.931	1.012
Age (years)	−0.002	0.004	−0.074	−0.010, 0.005	−0.643	0.522	1.214
ILR_1_[log(oxi/(red +roxi))]	0.075	0.101	0.079	−0.126, 0.275	0.740	0.462	1.054
ILR_2_[log(red/roxi)]	−0.300	0.073	−0.479	−0.447, −0.154	−4.091	**4.00 × 10^−4^**	1.260

B, unstandardized regression coefficient; SE, standard error; β, standardized regression coefficient; CI, confidence interval; VIF, variance inflation factor. The model was adjusted for age and sex. Bold values indicate statistical significance (*P* <0.05).

### Disease-specific redox profiles: neurodegenerative disorders versus other conditions

We investigated disease-related variations in CSF redox-IDX-apoE in patients with apoE3/E3. From this cohort, 20 patients with tumors or tumor-related disorders (G5) were excluded from the analysis. As these cases were predominantly non-neurological hematologic malignancies and involved mainly younger individuals, their inclusion could have introduced confounding factors related to both age distribution and disease characteristics. Consequently, a comparison was performed among the remaining four diagnostic groups (G1, G2, G3, and G4) (see Table 1 for demographics and Supplementary Table S1 for specific diagnoses).

**Figure 5:**
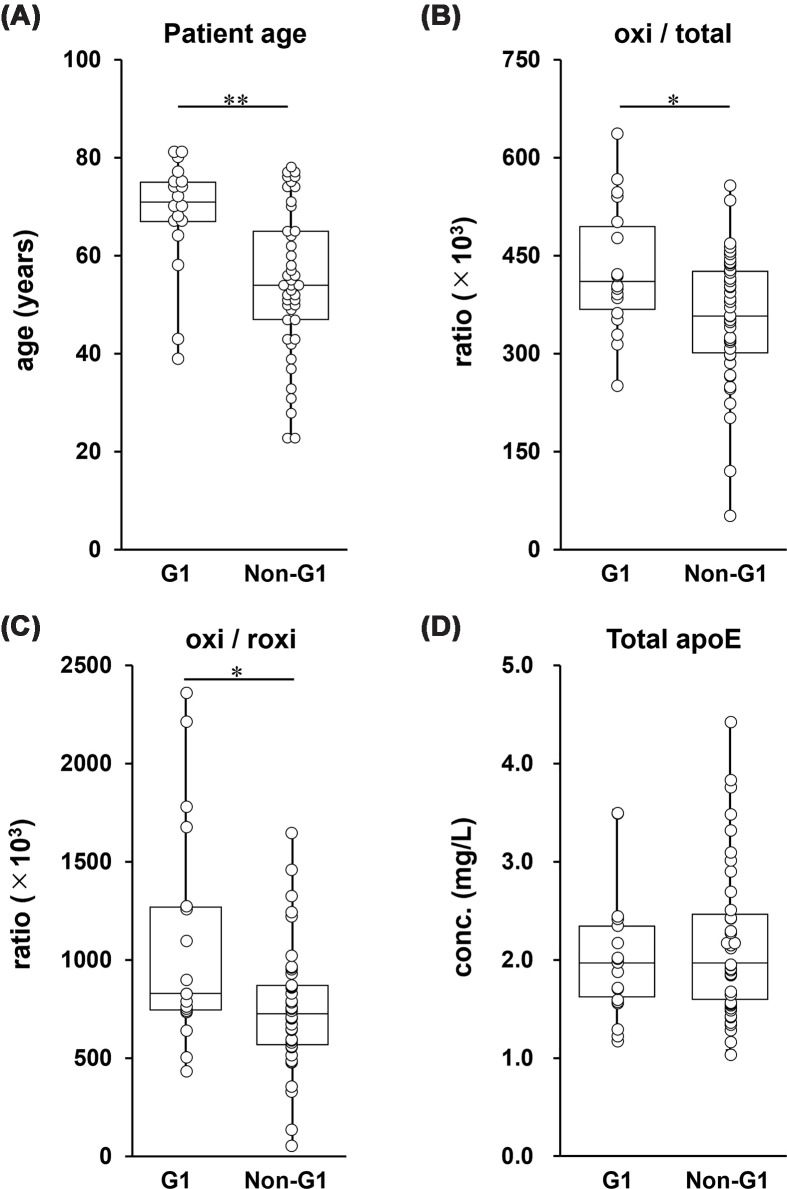
Comparison of age, total CSF apoE concentration, and redox indices (oxi/total and oxi/roxi) between neurodegenerative (G1) and non-neurodegenerative (non-G1) groups in apoE3/E3 subjects Boxplots showing age (**A**), oxi/total (**B**), oxi/roxi (**C**), and total CSF apoE concentration (**D**) in apoE3/E3 subjects comparing the neurodegenerative group (G1) with the combined non-neurodegenerative group (non-G1; G2 + G3 + G4). Tumor-related disorders (G5) were excluded from this analysis. Data are presented as box-and-whisker plots (medians and interquartile ranges) with individual data points overlaid (jittered). Statistical differences were analyzed using the Mann–Whitney U test. *P <0.05, **P <0.01.

To assess distinct pathological signatures, the neurodegenerative group (G1) was compared with the combined non-G1 group (G2 + G3 + G4). To facilitate interpretation, we focused primarily on oxi-related indices, because these provided the clearest disease-associated differences between the two groups. Statistical analysis revealed significant differences in the specific redox indices (Table 6 and Figure 5). Although the age of the G1 group was significantly higher than that of the non-G1 group (*P* <0.01), the G1 group exhibited significantly higher oxi/total and oxi/roxi values (*P* <0.05). No significant differences were observed in the other indices. Crucially, total CSF apoE concentrations remained comparable across diagnostic groups.

**Table 6:** Comparison of CSF apoE redox indices between neurodegenerative (G1) and non-neurodegenerative (Non-G1) groups in apoE3/E3 subjects

Variables	G1	Non-G1	*P*
Demographics			
n (female/male)	18 (10/8)	42 (26/6)	0.0521
age	68.6 ± 12.1	54.7 ± 14.8	**0.0018**
Redox indices			
red/total (×10^−3^)	114 (66–174)	127 (85–221)	0.2521
roxi/total (×10^−3^)	457 (344–532)	482 (408–557)	0.2727
oxi/total (×10^−3^)	411 (368–495)	358 (301–426)	**0.0249**
oxi/red (×10^−3^)	3477 (2622–7807)	3063 (1522–4629)	0.1557
oxi/roxi (×10^−3^)	829 (746–1269)	727 (569–871)	**0.0307**
red/roxi (×10^−3^)	279 (112–455)	221 (154–467)	0.9101
oxi/(red + roxi) (×10^−3^)	659 (504–865)	573 (450–780)	0.4293
red/(oxi + roxi) (×10^−3^)	129 (71–211)	120 (86–238)	0.6284
roxi/(red + oxi) (×10^−3^)	869 (526–1194)	851 (569–1029)	0.7106
Total apoE (mg/l)	1.97 (1.62–2.34)	1.97 (1.60–2.47)	0.7654

Data are presented as mean ± SD or median (interquartile range). G1, neurodegenerative diseases; Non-G1, combined group of neuroimmunological/inflammatory disorders, infectious diseases, and other conditions, excluding tumor-related disorders. Bold values indicate statistical significance (*P* <0.05).

Overall, these results reveal a distinct disease-specific redox signature in neurodegenerative disorders, characterized by a predominant shift toward irreversible oxidation (oxi-apoE) that appears to be independent of both total apoE concentration and the physiological aging process.

### Identification and visualization of disease-specific redox signatures

**Figure 6:**
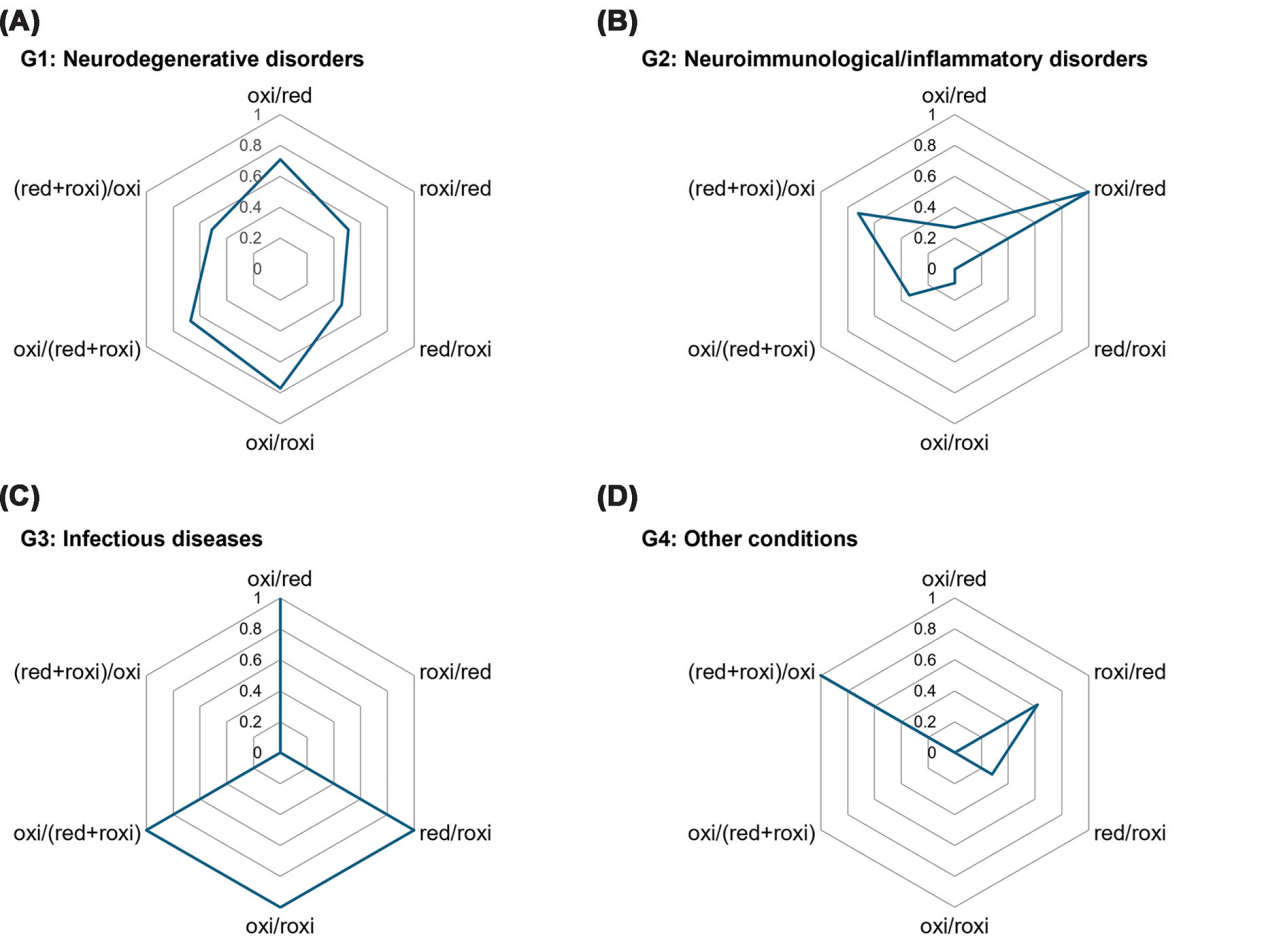
Disease-specific redox signatures of CSF apoE Radar chart illustrating the distinct redox profiles of the four diagnostic groups: G1 (neurodegenerative disorders) (**A**), G2 (neuroimmunological/inflammatory disorders) (**B**), G3 (infectious diseases) (**C**), and G4 (other conditions) (**D**). The axes represent the standardized mean values (*Z*-scores) of six key redox indices. Patients with tumor-related disorders were excluded from the analysis.

We performed a comprehensive four-group comparison to characterize the redox profiles of the neurodegenerative disorder group (G1) in comparison with those of the other diagnostic groups (G2–G4). Significant overall differences were observed across redox indices (Table 7). Although the average age of the G1 group was significantly higher than that of the G2 (*P* <0.01) and G3 groups (*P* <0.05), pairwise comparisons revealed the following disease-specific characteristics: (i) the G1 group exhibited significantly higher values than the G2 group in oxi/total, oxi/red, and oxi/roxi ratios (*P* <0.05); (ii) the G1 group also showed a significantly higher oxi/roxi ratio than the G4 group (*P* <0.05). To summarize the group-level differences in redox-IDX-apoE, the standardized mean values of the six redox indices were plotted on a radar chart (Figure 6). The chart highlights the distinct redox configurations for each diagnostic group. The G1 group displayed a broadly expanded pattern, particularly along the axes associated with irreversible oxidation, such as oxi/red and oxi/(red + roxi). The G2 group showed a prominent extension along the roxi/red axis, G3 exhibited a distinct spike along the oxi/roxi axis, and G4 showed the most contracted and centrally clustered profile. Despite these marked differences in redox signatures, the TC/apoE ratio did not differ significantly among the four groups (Supplementary Table S5).

**Table 7:** Comparison of CSF apoE redox profiles among four diagnostic groups in apoE3/E3 subjects

Redox indices	G1 (*n* = 18)	G2 (*n* = 22)	G3 (*n* = 7)	G4 (*n* = 13)	*P*
age	68.6 ± 12.1	51.8 ± 16.7	51.9 ± 16.6	61.2 ± 8.9	**0.0027** (G1 versus G2)
					**0.0469** (G1 versus G3)
Redox indices					
red/total (×10^−3^)	114 (66–174)	162 (92–230)	104 (74–145)	121 (79–190)	NS
roxi/total (×10^−3^)	457 (344–532)	482 (389–535)	485 (384–501)	484 (446–574)	NS
oxi/total (×10^−3^)	411 (368–495)	357 (299–411)	349 (308–415)	369 (313–429)	**0.0128** (G1 versus G2)
oxi/red (×10^−3^)	3477 (2622–7807)	2610 (1235–3744)	2669 (1554–3629)	3733 (1803–5795)	**0.0249** (G1 versus G2)
oxi/roxi (×10^−3^)	829 (746–1269)	743 (576–849)	706 (519–872)	731 (587–915)	**0.0112** (G1 versus G2)
					**0.0286** (G1 versus G4)
red/ roxi (×10^−3^)	279 (112–455)	274 (176–542)	207 (171–296)	205 (123–393)	NS
oxi/(red + roxi) (×10^−3^)	659 (504–865)	573 (461–718)	687 (468–1249)	585(456–752)	NS
red/(oxi + roxi) (×10^−3^))	129 (71–211	120 (93–268)	123 (90–260)	109 (64–231)	NS
roxi/(red + oxi) (×10^−3^)	869 (526–1194)	771 (576–1020)	908 (481–994)	866 (689–1170)	NS

Data are presented as median (interquartile range). G1, neurodegenerative diseases; G2, neuroimmunological/inflammatory disorders; G3, infectious diseases; G4, other conditions. NS, not significant (*P* >0.05).

## Discussion

Plasma and CSF apoE originate from distinct metabolic pools; the former is primarily synthesized in the liver, whereas the latter is independently produced in the CNS [1,3]. This independence is strikingly illustrated by the observation that liver transplantation alters the serum apoE phenotype of the recipient to that of the donor, whereas the CSF apoE phenotype retains the original genotype of the recipient [35]. Consistent with this biological compartmentalization, we observed significant but only moderate correlations between serum and CSF redox-IDX-apoE. This finding suggests that the systemic circulation and the CNS maintain largely independent redox environments with differing susceptibilities to oxidative stress. Although age-related oxidative stress may affect both systems universally, the redox-IDX of CSF apoE exhibited a more pronounced age-dependent shift than serum apoE. This implies that the CNS milieu may be more sensitive to or less capable of buffering cumulative oxidative stress. Furthermore, structural differences may also contribute to this divergence. Specifically, as confirmed by the results of neuraminidase digestion, CSF apoE undergoes a higher degree of sialylation than its plasma counterpart [34], potentially influencing its redox reactivity. Taken together, these observations underscore the importance of characterizing CSF-specific redox-IDX-apoE to elucidate the distinct redox regulation mechanisms and oxidative stress responses in the CNS.

Different apoE phenotypes also exhibited varied redox status of CSF apoE. Total apoE levels did not differ significantly among phenotype groups. However, red-apoE and roxi-apoE levels decreased in parallel with a reduction in the total number of Cys residues, with the apoE3/E4 phenotype showing the lowest levels. These findings are consistent with data from the serum reported in our previous study [16] and indicate that the combined abundance of red-apoE and roxi-apoE largely reflects the number of Cys residues available to participate in redox reactions. In contrast, the oxi-apoE levels (i.e., the PEG-PC-Mal–unconjugated monomeric fraction under the present assay conditions) were significantly higher in the apoE3/E4 phenotype than in the apoE3/E3 and apoE2/E3 phenotypes. This elevation, together with the reduced roxi-apoE levels, resulted in markedly higher oxi-based ratios (oxi/red, oxi/roxi, and oxi/(red + roxi)) and lower red- and roxi-based ratios in the apoE3/E4 phenotype. These redox profiles suggest that the apoE3/E4 phenotype has a reduced capacity for reversible oxidation and formation of disulfide-linked complexes, consistent with the lower availability of reactive Cys residues [36]. Importantly, because the oxi-apoE fraction corresponds to the non-conjugated fraction in the PEG-PC-Mal assay, phenotype-dependent differences in this index should be interpreted with caution and may partly reflect intrinsic isoform differences in cysteine content (particularly the presence of apoE4), in addition to oxidative modification status. Considering that complexes such as apoE homodimers and apoE–AII complexes buffer oxidative stress [14], the relative deficiency of these reversible forms in the apoE3/E4 phenotype may render it more susceptible to oxidative injury. Conversely, the apoE3/E3 and apoE2/E3 phenotypes, which possess a greater total number of Cys residues than apoE3/E4, exhibited lower oxi-apoE levels and higher red/(oxi + roxi) and roxi/(red + oxi) ratios. The absence of significant differences between apoE3/E3 and apoE2/E3 suggests that the Cys number alone cannot fully explain the observed variations. Rather, these findings imply that additional factors, such as isoform-specific structural or conformational differences, may contribute to phenotype-dependent redox behavior. Although a higher oxi-apoE index may be expected in phenotypes containing apoE4, which lacks Cys residues, the weaker dependency of CSF apoE redox status on Cys number compared with that of serum [16] is noteworthy. This discrepancy may primarily reflect differences in study populations: while our previous serum study examined individuals without disease [16], the present CSF analysis included patients with various clinical conditions that likely influenced the oxidative environment within the CNS.

Phenotype-dependent differences in apoE redox indices indicated that apoE isoforms differ in their redox behavior; therefore, the aging analysis was restricted to participants with the apoE3/E3 phenotype. With advancing age, CSF apoE exhibited characteristic shifts in redox balance. The total apoE concentration, as well as the roxi/total and oxi/red ratios, increased with age, whereas the red/total, red/roxi, and oxi/roxi ratios decreased, indicating that apoE molecules in the CNS of aged individuals become more susceptible to oxidative modification and less capable of sustaining reversible redox buffering. These findings suggest a progressive shift in the apoE redox equilibrium toward irreversible oxidation over an individual’s lifespan. The decline in oxi/roxi with age may appear paradoxical, as enhanced oxidative stress is expected to increase this ratio [30]. A plausible explanation for this is a biphasic change in reversible oxidation during aging. In early and mid-adulthood, increased formation of roxi-apoE, possibly via apoE–AII complex formation, may occur as a compensatory response to increased oxidative stress. Because apoAII is not synthesized in the CNS [37] and must be supplied via systemic circulation, roxi-apoE formation depends partly on peripheral apoAII availability. This mechanism may underlie the observed bell-shaped pattern of roxi-apoE: low in young individuals, peaking in middle-aged individuals when apoAII availability and blood–brain barrier permeability may better support complex formation [38], and declining in older age when oxidative stress becomes dominant [30], exceeding the buffering capacity of reversible thiol–disulfide exchange. This interpretation is further supported by the roxi/(red + oxi) ratio, which displayed a nonlinear, bell-shaped pattern, highest in middle-aged individuals and lower in both younger and older individuals, supported by our data. Notably, the oxi/(red + roxi) ratio, a representative index of irreversible oxidation, did not show a clear age-dependent increase. This may be partially explained by the bell-shaped increase in roxi-apoE, which could mask an underlying increase in irreversible oxidation. Our previous *in vitro* studies demonstrated that disulfide-linked apoE complexes, including apoE homodimers and apoE–AII complexes, buffer against oxidative stress and prevent irreversible apoE oxidation [14]. Therefore, we speculate that similar reversible oxidation mechanisms operate *in vivo* within the CNS to preserve apoE function against age-related oxidative stress.

Cholesterol is an essential lipid in the CNS required for synapse formation, membrane integrity, and myelin maintenance, and is largely synthesized *de novo* within the brain [39,40]. Correlations between CSF TC and redox-IDX-apoE levels further support the functional relevance of redox modulation. CSF TC levels correlated positively with total apoE, roxi/total, and roxi/(red + oxi) but negatively with oxi/total, red/roxi, oxi/roxi, and oxi/(red + roxi). Similarly, the TC/apoE ratio, an inverse indicator of apoE lipid transport efficiency, was positively correlated with oxi/red and roxi/(red + oxi), whereas red/roxi was negatively correlated. These findings suggest that maintaining apoE in its reduced monomeric state (red-apoE) is crucial for optimal lipid transport, whereas the shift toward oxidation, whether reversible or irreversible, is correlated with reduced efficiency. Overall, these results indicate that CNS cholesterol homeostasis depends not only on apoE abundance but also on its redox balance. Although evidence from earlier studies suggests that apoE homodimers (roxi-apoE) may enhance cholesterol efflux [41], our *in vivo* studies imply that the transition from a reduced monomer to an oxidized dimer comes at a functional cost. A likely mechanism involves a balance between lipid loading (efflux) and clearance (uptake). Although roxi-apoE may effectively accept lipids [41], disulfide-linked dimers exhibit markedly reduced affinity for the low-density lipoprotein receptor compared with monomers [26,42]. This impairment of receptor-mediated uptake would lead to the accumulation of cholesterol-loaded apoE in the CSF, thereby elevating the TC/apoE ratio (lower clearance efficiency) associated with roxi-based indices. Thus, although reversible oxidation acts as a chemical buffer against oxidative stress [14], a mechanism supported by the age-dependent “bell-shaped” rise in specific roxi indices described above, this protection appears to be a functional trade-off that compromises lipid transport capacity.

For a better understanding of the independent drivers of these functional associations, we performed a multivariate ILR regression analysis to identify the apoE redox parameters that independently influence apoE-mediated lipid transport. Consistent with the univariate correlations, ILR_2_ [log(red/roxi)], which represents the balance between the reduced state and the reversibly oxidized pool, was independently and negatively associated with the TC/apoE ratio. This finding reinforces the conclusion that maintaining apoE in its reduced monomeric form, rather than in its reversibly oxidized form, supports optimal cholesterol transport efficiency. The consistency between the univariate correlations and results of the multivariate ILR supports the robustness of this relationship. These findings substantiate our hypothesis that the oxidative state of apoE directly modulates its lipid transport capacity and that the transition from the reduced monomer to oxidized forms, even if reversible, marks a functional decline. Although the only evidence of the redox mechanisms of apoE is our previous study on serum apoE [16], the present *in vivo* data indicate that similar reversible oxidative regulatory processes operate within the CNS. Such mechanisms likely represent a physiological trade-off—sacrificing lipid transport efficiency to prioritize antioxidant buffering against oxidative stress. Notably, the redox indices that best predicted cholesterol transport efficiency did not fully overlap with those that exhibited the most pronounced disease-specific alterations. This divergence likely reflects the multilayered nature of apoE redox biology—early and reversible oxidation states (shift to roxi-apoE) primarily influence the lipid transport function and represent a compensatory phase, whereas chronic accumulation of irreversible oxidation more strongly reflects disease-specific oxidative environments. Thus, CSF redox-IDX-apoE captures distinct but complementary biological dimensions—functional impairment on one hand and pathological oxidative context on the other—highlighting their combined potential as mechanistically informative biomarkers.

Beyond physiological modifiers such as apoE isoforms and aging, the present study demonstrates that pathological conditions exert distinct and disease-specific influences on the apoE redox status within the CNS. Importantly, our findings demonstrate that these alterations are independent of total apoE levels. This suggests that the pathological significance of apoE in these conditions lies not in its abundance, but in its qualitative redox state. Each diagnostic category displayed a characteristic redox signature, indicating that the prevailing oxidative environment differentially shaped the distribution of reduced, reversibly oxidized, and irreversibly oxidized apoE species. Neurodegenerative disorders (G1) were marked by a pronounced shift toward irreversible oxidation, indicated by significantly elevated oxi/total and oxi/roxi ratios compared with other diagnostic groups. This configuration contradicts the physiological aging trajectory. As shown in our age-stratified analysis, the oxi/total ratio remained stable with age, whereas the oxi/roxi ratio significantly decreased among aged individuals. The fact that the G1 group, despite comprising individuals of a significantly higher age, exhibited elevated values for both indices strongly suggests that these alterations are not merely bystanders of senescence but are driven by disease-specific pathology. This configuration aligns with the chronic oxidative burden in neurodegenerative disorders [43–45] and suggests a heightened vulnerability of apoE redox homeostasis that exceeds the baseline oxidative shifts of aging. In contrast, the neuroimmunological and inflammatory disorders group (G2) exhibited significantly lower levels of irreversible oxidation indices (oxi/total, oxi/red, and oxi/roxi) than the neurodegenerative group (G1). This indicates that, unlike the pattern observed in neurodegeneration, oxidative modifications in these inflammatory conditions remain largely reversible. The predominance of the reversible redox potential (as suggested by the radar chart profile) is conceptually in line with the redox-dependent thiol–disulfide exchange processes during immune activation that have been previously described [46] and contrasts with the irreversible oxidative trajectory observed in the G1 group. Although pairwise comparisons did not indicate statistical significance, infectious diseases (G3) exhibited a distinct visual pattern on the radar chart, appearing to lean toward elevated oxi/roxi ratios. This unique profile suggests rapid and preferential conversion of apoE to its irreversibly oxidized form in specific cases. This pattern likely reflects acute oxidative surges triggered by infectious inflammation [47], which can transiently exceed the reversible redox buffering capacity. The heterogeneous G4 group, composed mainly of individuals with non-neurological conditions who did not meet the criteria for G1–G3, consistently showed the lowest oxidative indices, suggesting minimal perturbation of the CNS redox equilibrium. Collectively, these disease-related configurations indicate that the apoE redox state functions not only as a downstream marker of oxidative stress, but also as an index of disease-specific redox perturbations within the CNS. Notably, despite clear differences in the redox architecture across diagnostic groups, the TC/apoE ratio did not vary significantly among them. While our regression analysis identified apoE redox status as a key determinant of lipid transport efficiency, the stability of the TC/apoE ratio across diagnostic groups suggests that compensatory mechanisms or other disease-related factors (e.g., altered cholesterol synthesis or neuronal loss) may mask these redox-driven functional shifts at the group level. This observation implies that CSF redox-IDX-apoE captures subtle underlying qualitative alterations in the oxidative milieu that precede or exist independently of overt failure in bulk cholesterol homeostasis, reinforcing its value as a mechanistically informative biomarker for characterizing CNS disease pathology.

The present study has several limitations. It was a single-center study with a modest sample size, particularly within certain diagnostic groups. The heterogeneity of underlying clinical conditions among CSF donors may also have introduced variability in CNS oxidative stress that could not be fully controlled. Furthermore, the cross-sectional design precluded inferences regarding the causality or temporal evolution of the apoE redox alterations. Larger, multicenter, longitudinal studies are required to validate the present findings and further elucidate the dynamic regulation of apoE redox biology within the CNS.

In conclusion, our findings demonstrate that the apoE redox state is a key regulator of its functional capacity in the CNS. Specifically, maintenance of the reduced monomeric form with preserved Cys-thiols appears to be critical for optimal cholesterol transport efficiency, whereas the shift toward oxidation, even if reversible, is associated with a functional cost. Moreover, each diagnostic group exhibited a distinct CSF redox-IDX-apoE profile, indicating that apoE redox status reflects the qualitative nature of CNS pathology. Taken together, these results highlight the potential significance of CSF redox-IDX-apoE as a mechanistically informative biomarker for evaluating both the pathological oxidative environment and the consequent functional impairment of apoE.

## Supplementary Material

Supplementary Figures S1-S3 and Tables S1-S5

## Data Availability

The data that support the findings of the present study are openly available from the corresponding author, [K.Y.], upon reasonable request.

## Abbreviations

AD: Alzheimer’s disease
apo: Apolipoprotein
Arg: arginine
CNS: central nervous system
CSF: cerebrospinal fluid
Cys: cysteine
HRP: Horseradish peroxidase
ILR: isometric log-ratio
PEG-PC-Mal: photocleavable maleimide-conjugated polyethylene glycol
SD: standard deviation
TC: total cholesterol
VIF: variance inflation factors
