# Supplementary material for: Redox status of apolipoprotein E in cerebrospinal fluid: a mechanistically informative biomarker for central nervous system disorders
Source: Biosci Rep. 2026 May 14;46(5):BSR20250388. doi: 10.1042/BSR20250388 (PMC13181357; doi:10.1042/BSR20250388)
Supplement: Supplementary Figures S1-S3 and Tables S1-S5 [file BSR-2025-0388_supp.pdf]

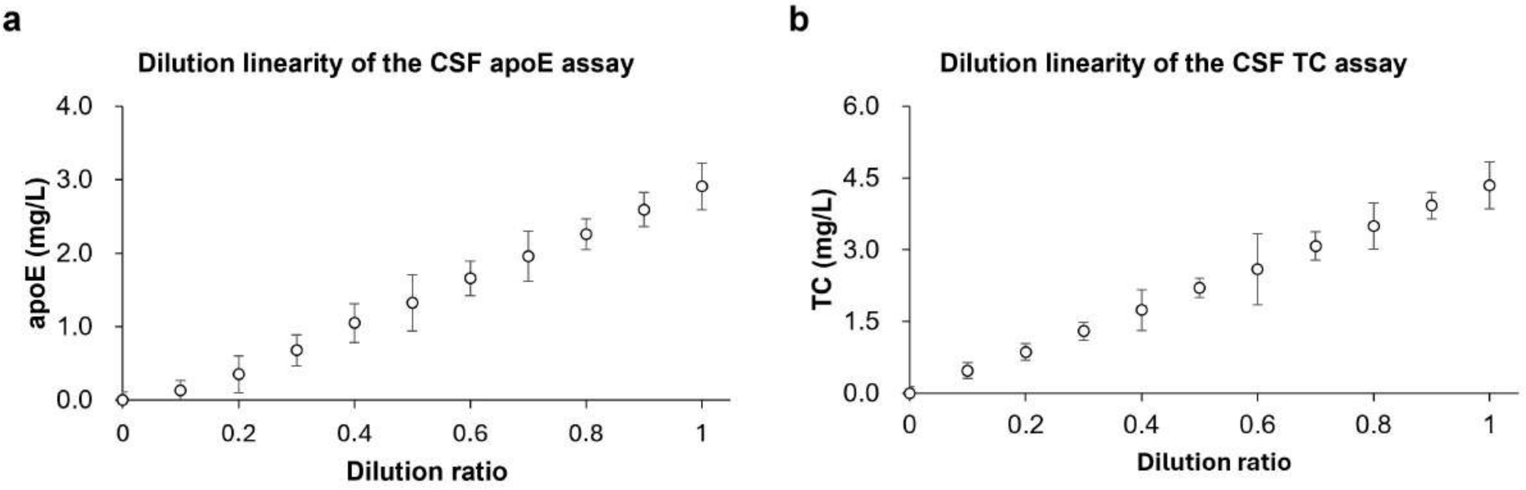

Supplementary Figure 1. Dilution linearity of the modified CSF apoE and total cholesterol (TC) assays.

Dilution linearity of the modified assays for CSF apoE (**a**) and TC (**b**) was evaluated using pooled CSF serially diluted with saline under the modified assay conditions used for CSF measurements in this study (20-fold increased sample-to-reagent volume ratio relative to the serum protocol). Each point represents the mean ± 2 SD from triplicate measurements.

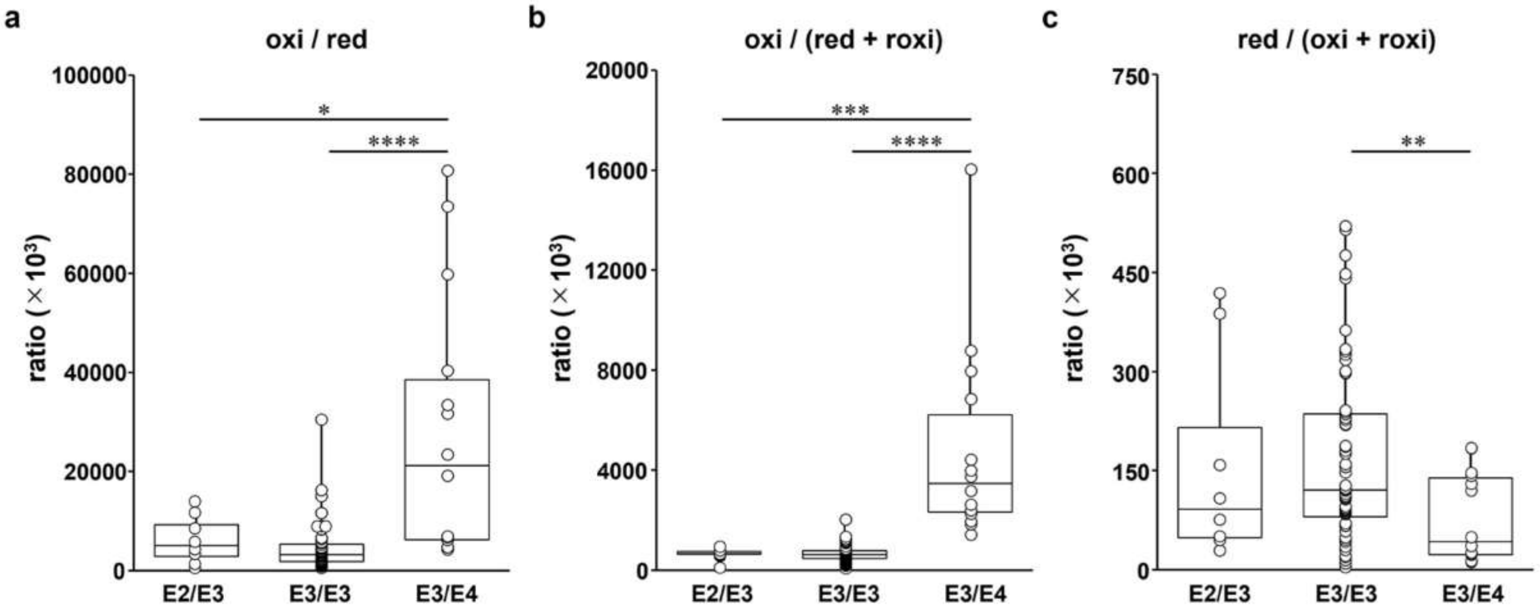

Supplementary Figure 2. Impact of apoE phenotype on redox indices in CSF.

Comparison of CSF apoE redox indices among participants with different phenotypes: E2/E3 (n = 8), E3/E3 (n = 80), and E3/E4 (n = 14). The ratios of oxi/red (**a**), oxi/(red + roxi) (**b**), and red/(oxi + roxi) (**c**) are shown. Data are presented as box-and-whisker plots (medians and interquartile ranges) with individual data points overlaid (jittered). Statistical differences were analyzed using the Kruskal–Wallis test, followed by the Steel–Dwass post-hoc test. *$p < 0.05$, **$p < 0.01$, *** $p < 0.001$, **** $p < 0.0001$.

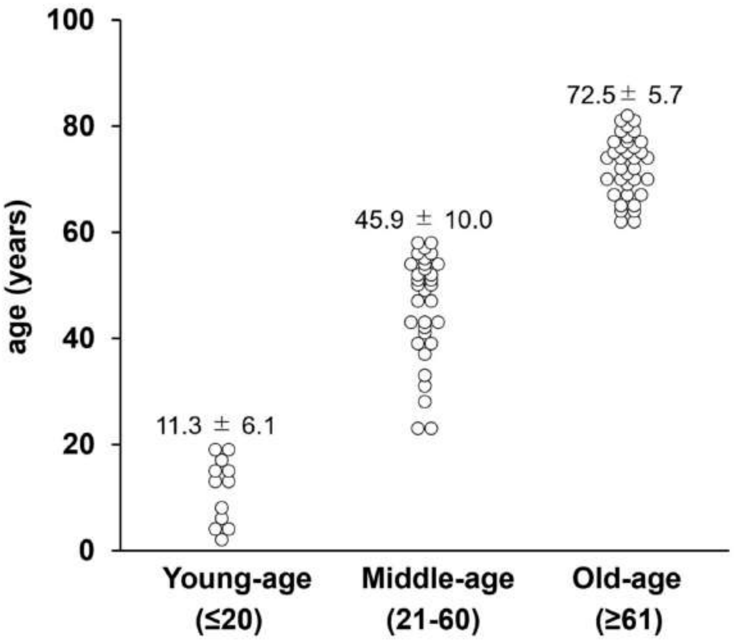

Supplementary Figure 3. **Individual age distribution of the age-defined apoE3/E3 cohorts.**
Individual ages of participants with the apoE3/E3 phenotype stratified into the young-age (≤20 years, n = 12), middle-age (21–60 years, n = 32), and old-age (≥61 years, n = 36) groups. Values above the plots indicate mean ± SD age for each group.

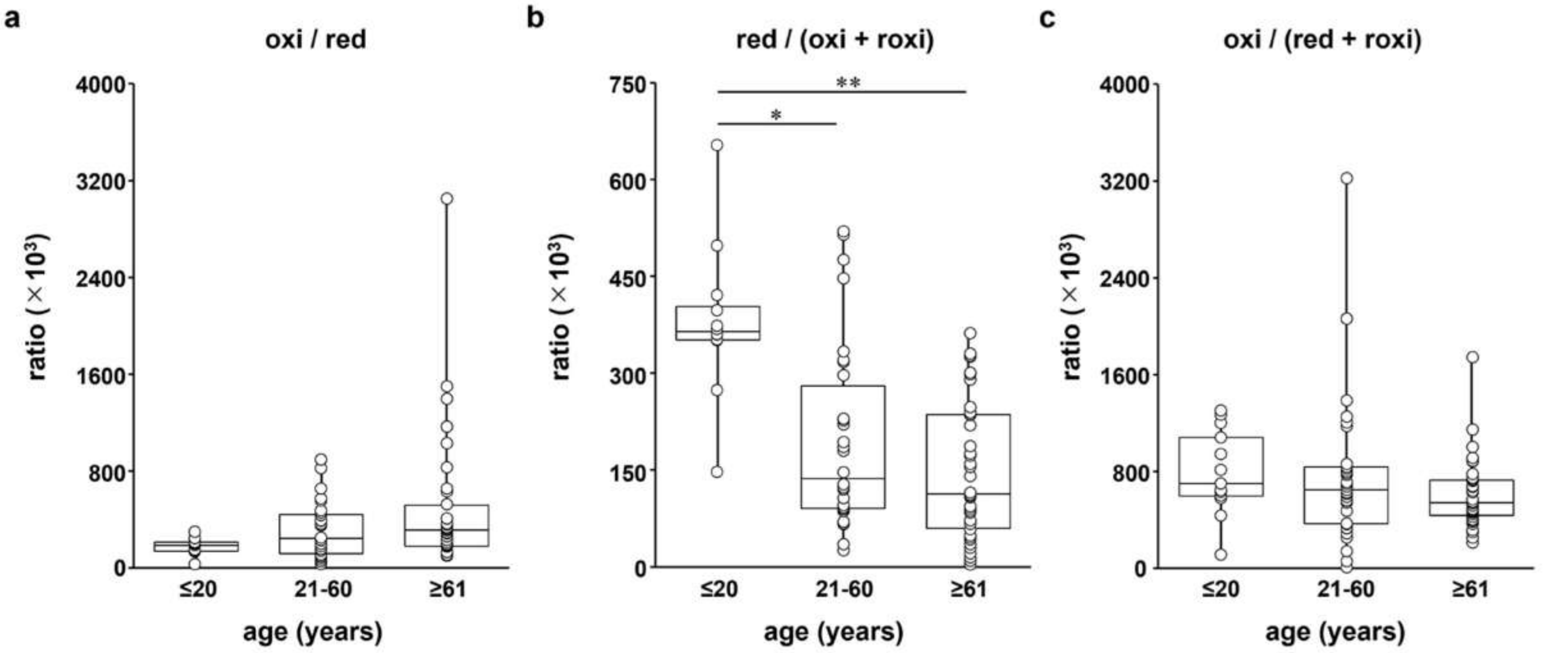

Supplementary Figure 4. Age-related changes in CSF apoE redox status in participants with apoE3/E3.

Participants with the apoE3/E3 phenotype were classified into three age groups: young-age (≤20 years, n = 12), middle-age (21–60 years, n = 32), and old-age (≥61 years, n = 36). The ratios of oxi/red (**a**), red/(oxi + roxi) (**b**), and oxi/(red + roxi) (**c**) are shown. Data are presented as box-and-whisker plots (medians and interquartile ranges) with individual data points overlaid (jittered). Statistical differences were analyzed using the Kruskal–Wallis test followed by the Steel–Dwass post hoc test. *$p < 0.05$, **$p < 0.01$.

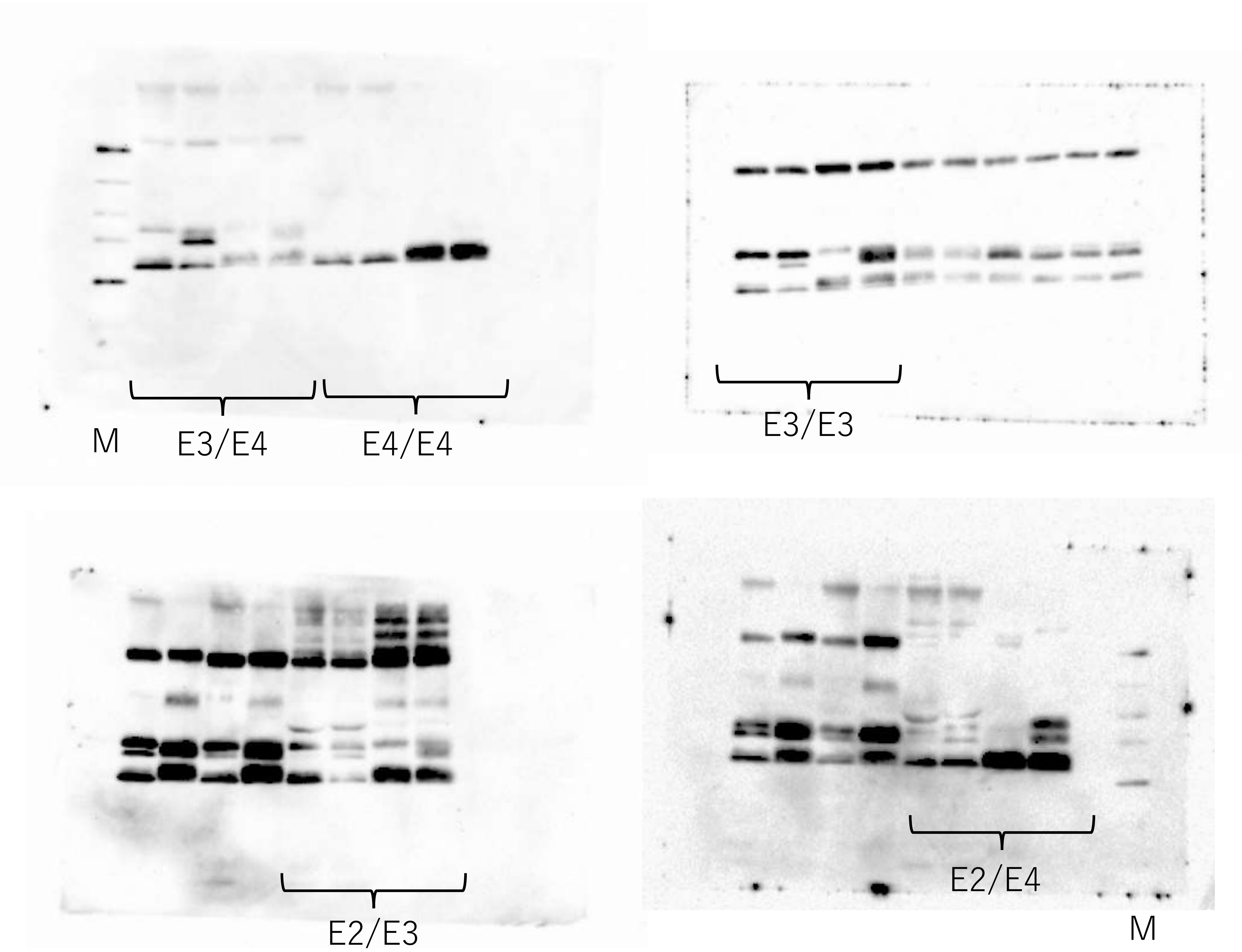

Original data of Figure 1a
We did not always load size markers because the molecular weights of the apoE monomer, homodimer, and E-AII complex are clearly defined.

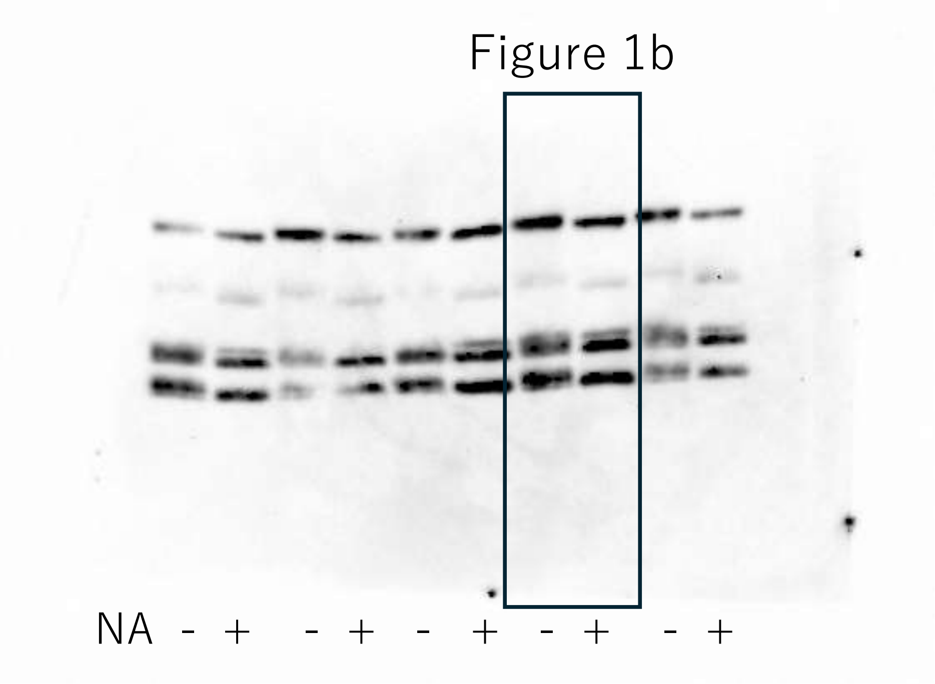

Original data of Figure 1b
We did not always load size markers because the molecular weights of the apoE monomer, homodimer, and E-AII complex are clearly defined.

Supplementary Table 1 Specific diagnoses and demographic characteristics of apoE3/E3 subjects included in the study

Diagnostic groups/Subcategory	n (female/male)	age	Specific diagnoses
**G1**: Neurodegenerative disorders	18 (10/8)	68.6 ± 12.1	
● Cognitive disorders	5 (2/3)	62.8 ± 15.4	AD, MCI, CAA
● Parkinsonian syndromes	6 (4/2)	73.4 ± 5.6	PD, MSA, Parkinsonism
● Motor neuron diseases & Others	7 (5/2)	69.4 ± 12.7	ALS, SMA type 4, SP
**G2**: Neuroimmunological/inflammatory disorders	20 (14/6)	51.8 ± 16.7	
● CNS demyelinating diseases &Encephalitis	7 (4/3)	46.1 ± 19.0	MS, GFAP astrocytopathy<br>Para-infectious AE
● PNS & Neuromuscular junction disorders	4 (3/1)	57.5 ± 11.5	Anti-MAG neuropathy, CIDP<br>myasthenia gravis
● Systemic autoimmune & Inflammatory disorders	9 (7/2)	52.4 ± 18.3	SLE, MCTD, GPA, MPA<br>Vogt-Koyanagi-Harada disease<br>Acute rheumatic fever
**G3**: Infectious diseases	7 (3/4)	51.9 ± 16.6	
● Meningitis & Encephalitis	2 (2/0)	49.0 ± 22.6	Aseptic meningitis, Syphilitic meningitis
● Other infections	5 (1/4)	53.0 ± 16.8	Neurosyphilis (meningitis, optic neuritis)

Table 1 continues on the next page.

**G4**: Other conditions	15 (7/8)	61.2 ± 8.9	

● Systemic & Metabolic conditions	2 (1/1)	63.5 ± 4.9	CHF, Delayed CO encephalopathy
● Other neurological disorders	13 (6/7)	60.8 ± 9.5	CS, Drug-induced dyskinesia, SM<br>Polyneuropathy, Epilepsy (TLE, NCSE)<br>CSS, spastic paresis, Distal arm weakness
**G5**: Tumor-related disorders<br>(Excluded from main analysis)	20 (9/11)	33.4 ± 30.8	
● Hematological malignancies	13 (5/8)	16.2 ± 18.5	ALL, AML, Malignant lymphoma
● Brain tumors	7 (4/3)	65.6 ± 21.6	Metastasis (melanoma, lung, thymic,<br>carcinomatous meningitis)<br>Meningioma, Pituitary adenoma<br>Schwannoma

AD, Alzheimer's disease; AE, autoimmune encephalitis; ALL, acute lymphoblastic leukemia; ALS, amyotrophic lateral sclerosis; AML, acute myeloid leukemia; CAA, cerebral amyloid angiopathy; CHF, congestive heart failure; CIDP, chronic inflammatory demyelinating polyneuropathy; CO, carbon monoxide; CS, cervical spondylosis; CSS, cavernous sinus syndrome; FND, functional neurological disorder; GFAP, glial fibrillary acidic protein; GPA, granulomatosis with polyangiitis; LGI1, leucine-rich glioma-inactivated 1; MAG, myelin-associated glycoprotein; MCI, mild cognitive impairment; MCTD, mixed connective tissue disease; MG, myasthenia gravis; MOG, myelin oligodendrocyte glycoprotein; MPA, microscopic polyangiitis; MS, multiple sclerosis; MSA, multiple system atrophy; NCSE, non-convulsive status epilepticus; PD, Parkinson's disease; SLE, systemic lupus erythematosus; SM, spinal myoclonus; SMA, spinal muscular atrophy; SP, spastic paraplegia; TLE, temporal lobe epilepsy

Supplementary Table 2 Within-run and between-run precision of the modified assays for CSF apoE and total cholesterol (TC)

	Sample	apoE		TC	
		mean (SD), mg/L	CV, %	mean (SD), mg/L	CV, %
Within-run (n = 20)	C1	2.65 (2.01×10^{-1})	7.6	4.36 (1.89×10^{-1})	4.3
	C2	0.50 (5.47×10^{-2})	10.9	0.89 (7.70×10^{-2})	8.7
Between-run (n = 10)	C1	2.47 (2.42×10^{-1})	9.8	4.52 (3.18×10^{-1})	7.0
	C2	0.44 (5.53×10^{-2})	12.6	0.87 (9.03×10^{-2})	10.4

C1 and C2 indicate pooled CSF tested as an undiluted and a 5-fold diluted specimen, respectively. Data are presented as mean ± SD and coefficient of variation (CV, %).

Supplementary Table 3 Spearman's rank correlation coefficients of apoE redox indices between CSF and serum

Redox-IDX-apoE	ρ	*p*
red/total	0.491	**$9.01{\times}10^{-8}$**
roxi/total	0.478	**$2.10{\times}10^{-7}$**
oxi/total	0.577	**$2.20{\times}10^{-16}$**
oxi/red	0.572	**$8.09{\times}10^{-11}$**
oxi/roxi	0.439	**$2.31{\times}10^{-6}$**
red/roxi	0.357	**$1.53{\times}10^{-4}$**
oxi/(red + roxi)	0.544	**$1.52{\times}10^{-9}$**
red/(oxi + roxi)	0.558	**$2.42{\times}10^{-10}$**
roxi/(red + oxi)	0.443	**$1.85{\times}10^{-6}$**
Total apoE	-0.034	0.726

Bold values indicate statistical significance ($p < 0.05$).

Supplementary Table 4 Biochemical characteristics of the age-defined apoE3/E3 cohorts

Age group	n	TC (mg/L)	apoE (mg/L)			
			Total	red	roxi	oxi
Young-age (≤20 years)	12	2.77 (2.10–3.38)	1.64 (1.52–1.99)	0.41 (0.31–0.58)	0.60 (0.41–0.85)	0.61 (0.40–0.79)
Middle-age (21–60 years)	32	3.49 (2.87–4.53)	1.89 (1.53–2.21)	0.20 (0.18–0.40)*	0.79 (0.53–1.05)	0.71 (0.49–0.95)
Old-age (≥61 years)	36	3.64 (3.01–4.07)*	2.39 (2.00–3.13)**, ##	0.24 (0.11–0.60)	1.18 (0.91–1.80) **, ##	0.96 (0.66–1.49) *, #

Data are presented as median (interquartile range). *, p <0.05; **, p<0.01 (vs young-age group). #, p <0.05; ##, p<0.01 (vs middle-age group).

Supplementary Table 5 Comparison of the TC/apoE ratio among the diagnostic groups

Variables	G1 (n = 18)	G2 (n = 22)	G3 (n = 7)	G4 (n = 13)
TC (mg/L)	3.17 (2.63–3.41)	3.79 (2.93–5.22)	4.11 (3.30–4.97)	4.00 (3.30–4.84)
Total apoE (mg/L)	1.97 (1.62–2.34)	1.94 (1.60–2.19)	2.21 (1.59–3.75)	2.15 (1.84–2.69)
TC/apoE	1.48 (1.27–1.54)	1.94 (1.50–2.50)	1.96 (1.63–2.45)	2.04 (1.30–2.60)

Data are presented as median (interquartile range). G1, neurodegenerative diseases; G2, neuroimmunological/ inflammatory disorders; G3, infectious diseases; G4, other conditions.
